# Age-friendly care for older adults with substance use disorder

**DOI:** 10.1016/S2666-7568(23)00174-5

**Published:** 2023-10

**Authors:** Katie F Jones, Kimberly J Beiting, Mim Ari, Rossana Lau-Ng, Andrea J Landi, Lauren Kelly, Vassiliki Pravodelov, Benjamin H Han

**Affiliations:** New England Geriatric Research, Education and Clinical Center, Department of Medicine, Section of Palliative Care, VA Boston Healthcare Systems, Boston, MA 02130, USA (KFJ); Vanderbilt University Medical Center, Division of Geriatric Medicine, Nashville, TN, USA (KJB); Department of Medicine, Section of General Internal Medicine (MA), and Section of Geriatrics and Palliative Medicine Faculty, MacLean Center for Clinical Medical Ethics (AJL), University of Chicago, Chicago, IL, USA; Chobanian & Avedisian School of Medicine, Boston University, Boston, MA, USA (RL-N, VP); Brookdale Department of Geriatrics and Palliative Medicine, Icahn School of Medicine at Mount Sinai, New York, NY, USA (LK); Division of Geriatrics, Gerontology, and Palliative Care, University of California, San Diego, CA, USA (BHH)

Substance use disorder is an important and increasingly prevalent condition among older adults (ie, over the age of 65 years), and can no longer be considered primarily a disorder of younger populations.^1^ Age-related biopsychosocial changes, such as physiological changes and social isolation, can increase the risk of substance-related harms and might drive unhealthy substance use. Drug overdoses and deaths caused by overdose, including among older adults, are occurring at record-high rates in the USA, where deaths from overdose in older adults have tripled between 2002 and 2021,^2^ and disproportionately affect racially minoritised populations due to a dangerous supply of recreational drugs and to racial and socioeconomic inequities in the treatment of substance use disorder.^3^ Although awareness of the prevalence of unhealthy substance use and of its related harms is increasing, older adults are rarely screened for substance use or offered approaches to minimise substance-related harms.^4^

Adults can age with an existing substance use disorder or develop a new-onset substance use disorder in later life. The current worldwide cohort of older adults has had greater exposure to, and has a higher social acceptance of, psychoactive substances than earlier generations, which has been resulting in higher rates of substance use, both previous and lifelong. New-onset substance use disorder in older adults might be precipitated by mental illness, trauma, psychosocial stressors (eg, isolation, grief, loss, and chronic pain or other chronic symptoms), or exposure to prescription psychoactive medications. Older adults with substance use disorder face a disproportionate burden of drug-related harms due to age-related physiological changes and multimorbidity. Ageing affects every organ system, most notably the cardiovascular, renal, hepatic, musculoskeletal, and nervous systems. Due to the increased burden of chronic diseases in this population, older adults tend to be more vulnerable to the adverse side-effects of substances and to substance-related morbidity and mortality. The use of alcohol and benzodiazepines has been associated with an increased risk of falls, cognitive impairment, and delirium in older adults.^6^ Stimulants can be particularly lethal to older adults due to the high rates of cardiovascular disease in this population. In addition, substance use disorder in older people can also worsen, or precipitate an earlier onset of, conditions such as cognitive impairment and frailty.^6^ These age-related changes can complicate and exacerbate the reality that many older adults with substance use disorder have unmet primary and geriatric care needs.

Despite the increasing prevalence and high risk of substance-related harms in older people, less than a third of substance use disorder treatment programmes are tailored for this population group.^5^ As adults age with substance use disorder, treatment can become increasingly complex due to factors such as fragmented care, transportation barriers, multimorbidity, and cognitive impairment. Ageism, persistent stigma associated with a diagnosis of substance use disorder, and a paucity of clinician knowledge of evidence-based substance use disorder care contribute to inequitable health care and to disproportionate harms for older adults who spend increasingly more time in, and transitioning between, health-care settings, such as hospitals and post-acute care facilities.^6^

A movement to build age-friendly health systems and to provide evidence-based, high-quality care to older adults is already underway.^7^ However, more and deeper discussions are needed about how the care for older adults who use psychoactive substances, who would undoubtedly benefit from such care,^8^ fits within an age-friendly system. Age-friendly care, represented by five focus areas of getriatric care, or the 5Ms (ie, matters most, medications, mind, mobility, and multicomplexity), can be used to move away from the existing fragmented and suboptimal health-care system by developing more holistic care plans for adults ageing with substance use disorder (figure).^9,10^ Application of the 5Ms model of age-friendly care should occur at the individual and system levels. When prescribing treatment for an older adult with substance use disorder, a clinician should consider what matters to the patient (eg, less time spent at doctors’ appointments), medication safety (eg, dexterity and cognitive capacity to self-administer medications and manage drug–drug interactions resulting from polypharmacy), mental health (eg, mood disorders, cognitive impairment), mobility limitations (eg, recent falls; transportation to treatment clinics (ie, transportation after driving cessation), and treatment of co-occurring diagnoses that add to multicomplexity (eg, comorbid diagnoses that might affect treatment plans and medication options).

The combination of ageing and increasing use of psychoactive substances, including misuse of prescription psychotropic medications, creates a growing public health problem. Increasing numbers of older adults worldwide are at risk of harm from drug use in the context of age-associated physiological changes, social factors, increases in comorbidity, and polypharmacy. To improve the health of this vulnerable population, approaches to care tailored to older people must be developed and integrated with addiction treatment to build age-friendly health systems that can address substance use disorder among older adults.

## Figures and Tables

**Figure:**
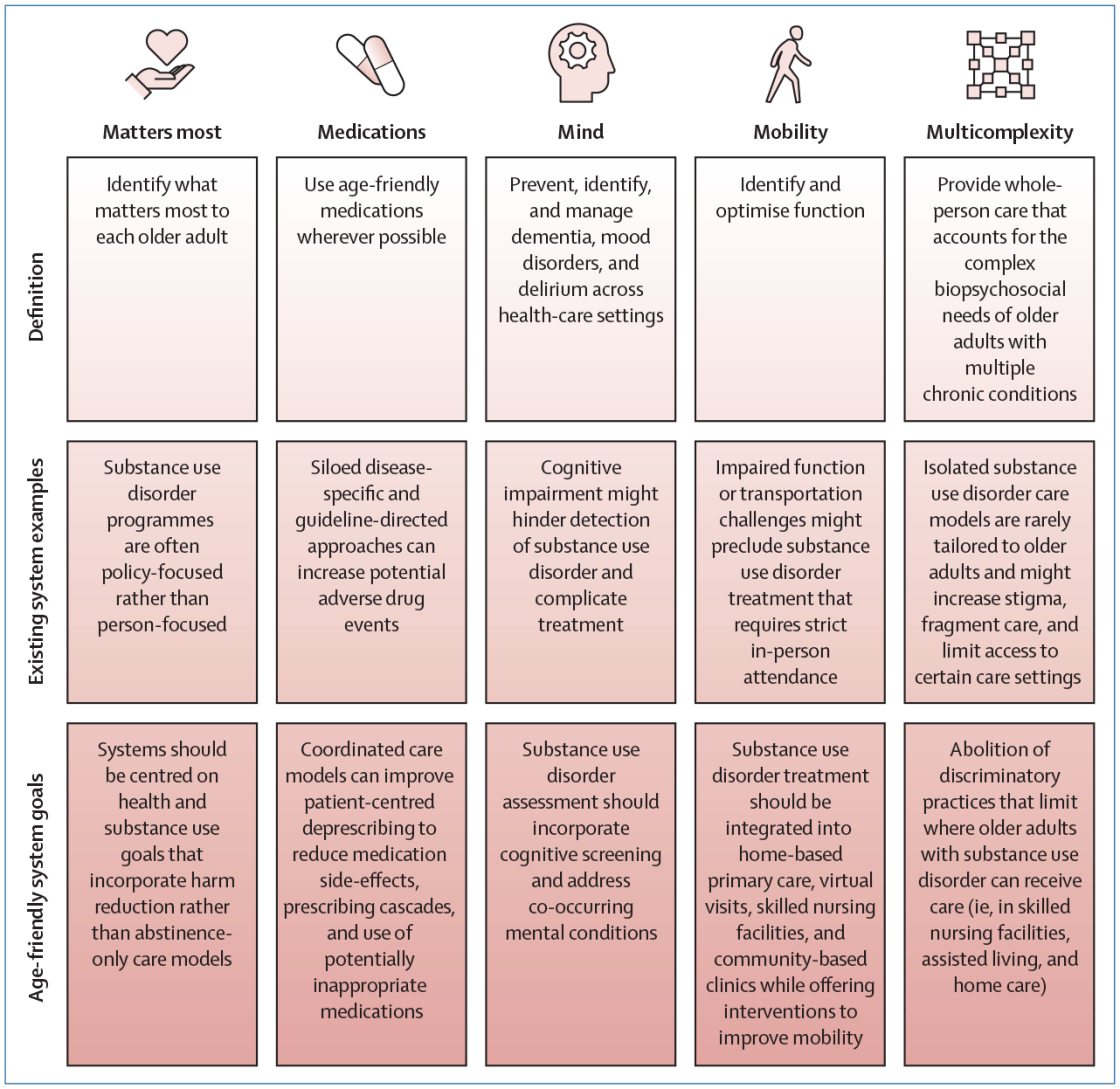
Reimagining an age-friendly substance use disorder health-care system
